# Antineoplastic Activity of Chrysin against Human Hepatocellular Carcinoma: New Insight on GPC3/SULF2 Axis and lncRNA-AF085935 Expression

**DOI:** 10.3390/ijms21207642

**Published:** 2020-10-15

**Authors:** Iman O. Sherif, Laila A. Al-Mutabagani, Dina Sabry, Nehal M. Elsherbiny

**Affiliations:** 1 Emergency Hospital, Faculty of Medicine, Mansoura University, Mansoura 35516, Egypt; 2 Chemistry Department, College of Science, Princess Nourah Bint Abdulrahman University, Riyadh 11671, Saudi Arabia; laalmutbagani@pnu.edu.sa; 3 Medical Biochemistry and Molecular Biology Department, Faculty of Medicine, Cairo University, Cairo 11562, Egypt; dinasabry@kasralainy.edu.eg; 4 Medical Biochemistry and Molecular Biology Department, Faculty of Medicine, Badr University in Cairo, Badr City 11829, Egypt; 5 Biochemistry Department, Faculty of Pharmacy, Mansoura University, Mansoura 35516, Egypt; drnehal@hotmail.com; 6 Pharmaceutical Chemistry Department, Faculty of Pharmacy, University of Tabuk, Tabuk 71491, Saudi Arabia

**Keywords:** hepatocellular carcinoma, Chrysin, GPC3, SULF2, lncRNA-AF085935

## Abstract

The natural flavonoid chrysin possesses antiproliferative activity against various types of cancers, including hepatocellular carcinoma (HCC), which is a common malignancy. However, the exact mechanism of chrysin antiproliferative activity remains unclear. This research was executed to explore the impact of chrysin on glypican-3 (GPC3)/sulfatase-2 (SULF2) axis and lncRNA-AF085935 expression in HCC using HepG2 cells. Cisplatin (20, 50, 100 μg/mL), chrysin (15, 30, and 60 μg/mL) and the combination of 50 μg/mL cisplatin with different concentrations of chrysin were applied for 24/48 h. Cell viability was determined by MTT assay. Protein levels of GPC3 and SULF2 were measured by ELISA at 24/48 h. GPC3 immunoreactivity was detected by immunocytochemistry. Moreover, GPC3 and SULF2 mRNA expressions in addition to lncRNA-AF085935 expression were assessed by qPCR at 48 h. The GPC3 protein, immunostaining and mRNA levels, SULF2 protein and mRNA levels, as well as lncRNA-AF085935 expression, were decreased significantly with cisplatin and chrysin alone when compared with the control untreated HepG2 cells. However, the combination treatment exhibited a better chemopreventive effect in a dose- and time-dependent manner. This study demonstrated, for the first time, the antiproliferative activity of chrysin against HCC through the suppression of the GPC3/SULF2 axis along with the downregulation of lncRNA-AF085935 expression. Synergistic effect of chrysin with cisplatin could potentiate their antiproliferative action in a dose- and time-dependent manner.

## 1. Introduction

Liver cancer is a serious health problem and is considered the world’s main cause of cancer related death. The most prevalent type is known as hepatocellular carcinoma (HCC) [1]. Retarding the progression of HCC is the target plan for physicians to improve a patient’s prognosis [2]. Although physicians have tried their best to treat HCC through surgical operations, either by liver resection or liver transplantation, missing early diagnosis led to poor prognosis [1,3]. Unfortunately, most HCC patients are diagnosed at their advanced stages with systemic chemotherapies being the only treatment option [4].

Currently, the chemotherapeutic agents utilized for HCC are associated with deleterious adverse effects on their use [5]. Therefore, the use of natural products, either alone or in combination with conventional chemotherapy, is gaining great interest. Indeed, various studies applied natural products as sole treatment or combination regimens with common chemotherapy for the purpose of HCC treatment and/or reduction of the cytotoxicity of chemotherapy [6,7,8]. Cisplatin is one of the most effective and frequently used chemotherapeutic drugs for the management of HCC, with high response rates and favorable long-term outcomes [9]. Currently, multiple studies are focusing on developing novel combination regimens to potentiate cisplatin antiproliferative efficacy and facilitate its clinical application [10,11].

The natural polyphenolic compounds that occur in plants were known as flavonoids and possess various beneficial pharmacological actions including anti-inflammatory, antiapoptotic, antioxidants, and anticancer activities [12,13]. One of these natural flavonoids is chrysin, with the chemical structure of 5,7-dihydroxyflavone, which is present in propolis and honey [14]. The chrysin antitumor activity was documented in various types of human cancer cell lines, including gastric [15], breast [16], colorectal [17], and prostate [18] cancer cells due to its ability to induce cell apoptosis and cell cycle arrest [19]. Interestingly, previous studies showed the antitumor potential of chrysin against HCC through p53/Bcl-2/caspase-9 pathway activation [20] and targeting hexokinase-2 [12]. Notably, chrysin has been reported to alleviate cisplatin-induced liver [21] and colon [22] toxicity, highlighting its beneficial use as combinational therapy with cisplatin. However, the exact mechanism of chrysin anti-proliferative effect remains unidentified.

On the other hand, remodeling of the extracellular matrix (ECM) is significantly involved in the control of HCC differentiation, proliferation, and metastasis [23]. Heparan sulfate proteoglycans (HSPGs) are ECM cell surface macromolecules comprised of a core protein attached to chains of heparan sulfate glycosaminoglycan (HS GAG). Glypicans (GPCs) are cell surface HSPGs and are connected to the plasma membrane by glycosylphosphatidylinositol (GPI) link [24]. Glypican-3 (GPC3) acts as a storage site for some ligands through the HS side chain. GPC3 enables ligand/receptor interaction to stimulate various signaling pathways incorporated in the progression of the HCC [3]. 

The extracellular enzyme sulfatase 2 (SULF2) is involved in the tumorigenesis and progression of several carcinomas through alteration of sulfation on HSPGs. SULF2 is characterized as having a 6-O-desulftase activity, which removes the 6-O-sulfate from HSPGs, minimizing the affinity of HSPGs to sequester signaling ligands, leading to their release from the HSPGs storage sites and driving them accessible for activating various pathways [25,26].

Additionally, it was reported that GPC3 and SULF2 overexpression were associated with HCC growth [3,27,28]. GPC3 is currently considered as a trustable indicator for the HCC earlier diagnosis and prognosis than serum alpha-fetoprotein [3,27]. Moreover, SULF2 was reported to be a crucial indicator for the prognosis of HCC [28]. Beside their diagnostic value, a large body of evidence reported that targeting GPC3 and SULF2 is a promising therapeutic approach for HCC [29,30]. 

Likewise, long noncoding RNA (lncRNA), with >200 nucleotides length, have been recently emerged as gene expression regulators in cancer cells, including HCC [31]. Several lncRNAs are dysregulated in liver cancer cells and have been linked to crucial signaling pathways of tumorigenesis. These dysregulated lncRNAs are strongly suggested as novel diagnostic biomarkers and/or therapeutic targets for HCC [32]. Specifically, the lncRNA-AF085935 was highly expressed in HCC and could be used as a vital biomarker for HCC [33]. This study was carried out to discover a new insight of the antiproliferative action of chrysin alone or with its concurrent treatment with cisplatin against HCC through the impact on the GPC3/SULF2 axis in addition to lncRNA-AF085935 expression.

## 2. Results

### 2.1. Chrysin Combined with Cisplatin Decreased Cell Survival in HepG2 Cells

**Figure 1:**
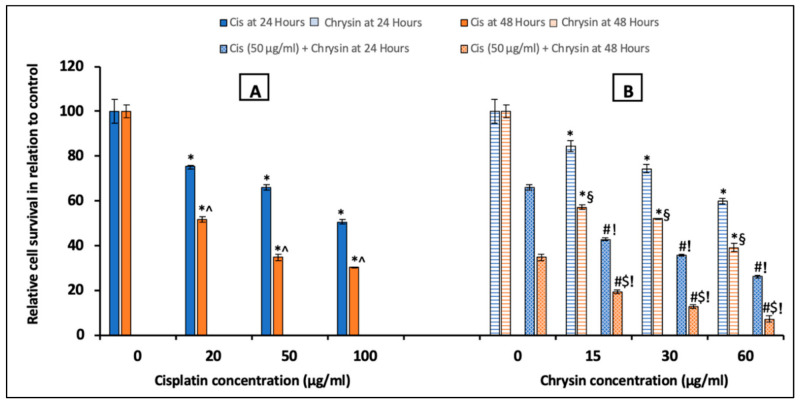
Impact of 24 and 48 h treatment with (**A**) cisplatin (Cis; 20, 50, 100 µg/mL), (**B**) chrysin (15, 30, 60 µg/mL) and combination of chrysin (15, 30, 60 µg/mL) with Cis 50 µg/mL on cell survival in HepG2 cells. Results are presented as Mean ± SD. * *p* < 0.05 versus the control untreated cells. ^^^ *p* < 0.05 versus the same dose of Cis treatment for 24 h. ^#^ *p* < 0.05 versus the same dose of chrysin at the same incubation time. ^!^ *p* < 0.05 versus Cis 50 µg/mL at the same incubation time. ^§^ *p* < 0.05 versus the same dose of chrysin treatment for 24 h.^$^ *p* < 0.05 versus cis 50 µg/mL + the same dose of chrysin treatment for 24 h.

The MTT assay was utilized to evaluate the impact of chrysin and cisplatin (alone or in combination) on HepG2 cells proliferation (Figure 1). Cisplatin (20, 50, 100 µg/mL) (A) and chrysin (15, 30, 60 µg/mL) (B) sole treatment for 24 and 48 h markedly reduced the survival of HepG2 cells in a concentration-dependent manner when compared with the control untreated HepG2 cells, (*p* < 0.05). The combination of chrysin (15–60 µg/mL) with cisplatin 50 µg/mL treatment for 24 and 48 h significantly reduced the HepG2 cells survival when compared with chrysin (15–60 µg/mL) alone or cisplatin 50 µg/mL for 24 and 48 h, respectively (*p* < 0.05, Figure 1B). Moreover, our results documented that the significant reduction in cell survival was obtained in a time-dependent way in which more reduction in cell survival was determined after 48 h treatment compared to 24 h treatment (*p* < 0.05, Figure 1).

### 2.2. Chrysin Combined with Cisplatin Decreased Glypican-3 (GPC3) Protein and mRNA Expressions in HepG2 Cells

**Figure 2:**
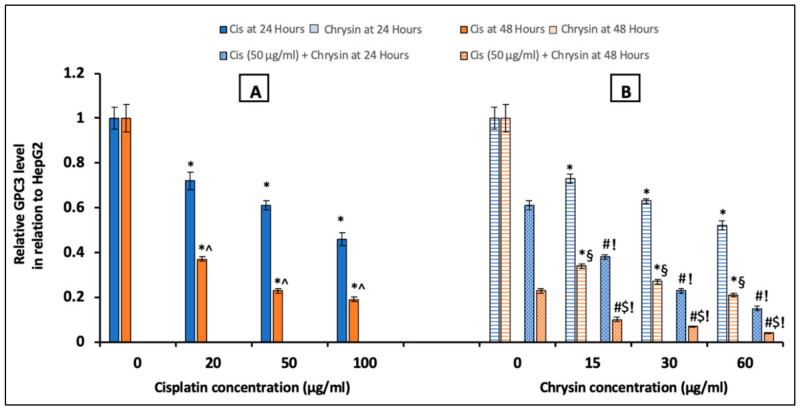
Impact of 24 and 48 h treatment with (**A**) cisplatin (Cis; 20, 50, 100 µg/mL), (**B**) chrysin (15, 30, 60 µg/mL) and combination of chrysin (15, 30, 60 µg/mL) with Cis 50 µg/mL on glypican-3 (GPC3) protein levels in HepG2 cells. Results are presented as Mean ± SD. * *p* < 0.05 versus the control untreated cells. ^^^ *p* < 0.05 versus the same dose of Cis treatment for 24 h. ^#^ *p* < 0.05 versus the same dose of chrysin at the same incubation time. ^!^ *p* < 0.05 versus Cis 50 µg/mL at the same incubation time. ^§^ *p* < 0.05 versus the same dose of chrysin treatment for 24 h. ^$^ *p* < 0.05 versus cis 50 µg/mL + the same dose of chrysin treatment for 24 h.

To further examine the molecular mechanism of the antiproliferative activity of chrysin on HepG2 cells, we assessed GPC3 protein levels by ELISA at 24/48 h and immunocytochemistry (ICC) at 48 h, in addition to its mRNA expression by qPCR at 48 h in cells treated with different concentrations of chrysin and/or cisplatin. Treatment with either cisplatin (20, 50, 100 µg/mL) (Figure 2A), or chrysin (15, 30, 60 µg/mL) (Figure 2B), notably reduced GPC3 protein levels after 24 and 48 h incubation in a concentration-dependent manner when compared with the control untreated HepG2 cells, (*p* < 0.05). 

**Figure 3:**
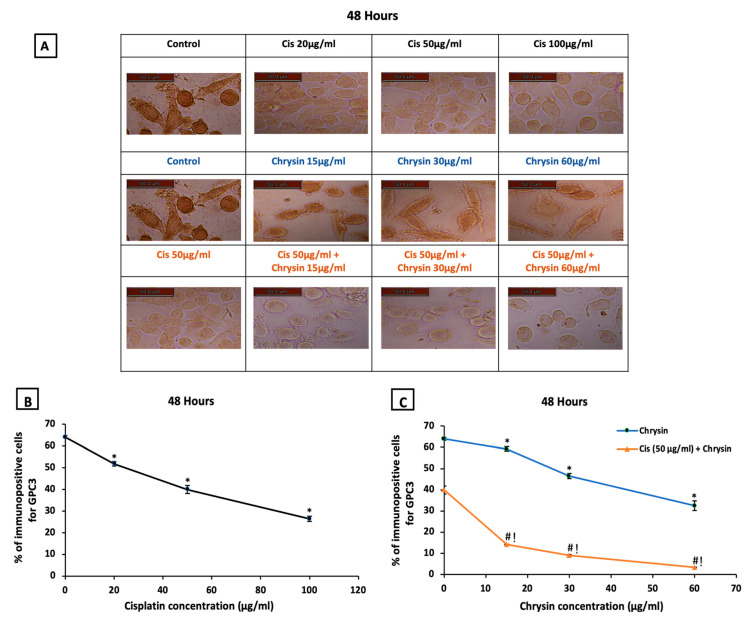
(**A**) Representative images of glypican-3 (GPC3) immuno-staining in HepG2 cells following treatments with cisplatin (Cis; 20, 50, 100 µg/mL), chrysin (15, 30, 60 µg/mL) and combination of chrysin (15, 30, 60 µg/mL) with Cis 50 µg/mL for 48 h, scale bar = 50 µm. Control untreated cells showed strong GPC3 immunopositive stain, Cis (20, 50, 100 µg/mL) treated cells showed mild GPC3 immunopositive stain, chrysin (15, 30, 60 µg/mL) treated cells showed GPC3 moderate immunopositive stain while, Cis 50 µg/mL + (15, 30, 60 µg/mL) treated cells showed weak GPC3 immunopositive stain. (**B**) Statistical analysis of the % of immunopositive cells for GPC3 after treatment with cisplatin (Cis; 20, 50, 100 µg/mL) for 48 h, (**C**) chrysin (15, 30, 60 µg/mL) as well as chrysin (15, 30, 60 µg/mL) and Cis 50 µg/mL combination for 48 h. * *p* < 0.05 versus the control untreated cells. ^#^ *p* < 0.05 versus the same dose of chrysin. ^!^ *p* < 0.05 versus Cis 50 µg/mL. Figures for the control group in the upper and middle lanes and for the Cis 50 µg/mL group in the upper and last lanes are identical to unify the reference groups in the statistical analysis.

The ICC for detection of GPC3 immunopositive cells at 48 h, presented in (Figure 3A), showed higher GPC3 protein expression in control untreated HepG2 cells. Mild GPC3 protein expression with cisplatin treatment and moderate GPC3 protein expression with chrysin sole treatment was observed in comparison to the control untreated cells. However, low GPC3 protein expression was observed in the combination-treated cells when compared to chrysin or cisplatin treated cells in a dose-dependent manner. 

Regarding the statistical analysis of GPC3 immunopositive cells detected by ICC, our results documented that the used sole treatments significantly reduced GPC3 immunostaining in HepG2 cells after 48 h incubation in a concentration-dependent manner when compared with control untreated HepG2 cells, (*p* < 0.05, Figure 3B,C).

Additionally, GPC3 protein levels (Figure 2B) and immunostaining (Figure 3C) in HepG2 cells were markedly reduced on the combination of chrysin (15–60 µg/mL) with cisplatin 50 µg/mL treatment when compared with chrysin (15–60 µg/mL) single treatment or cisplatin 50 µg/mL for the same incubation time (*p* < 0.05). Furthermore, our data showed that the GPC3 protein level was statistically non-significant in case of treatment with chrysin (15, 30 µg/mL) alone for 48 h when compared with chrysin (15, 30 µg/mL) combined with cisplatin 50 µg/mL for 24 h. However, more decline in the GPC3 protein expression was observed when using chrysin (15–60 µg/mL) combined with cisplatin 50 µg/mL for 48 h compared to 24 h, (*p* < 0.05, Figure 2B).

**Figure 4:**
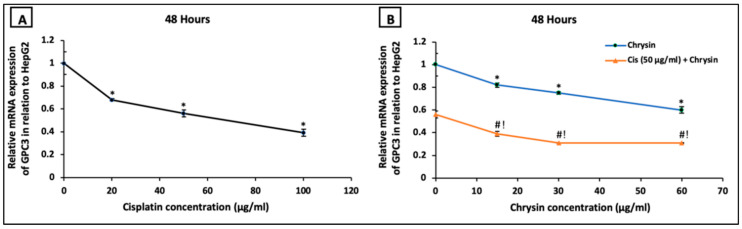
Impact of 48 h treatment with (**A**) cisplatin (Cis; 20, 50, 100 µg/mL), (**B**) chrysin (15, 30, 60 µg/mL) and combination of chrysin (15, 30, 60 µg/mL) with Cis 50 µg/mL on glypican-3 (GPC3) mRNA expressions in HepG2 cells. Results are presented as Mean ± SD. * *p* < 0.05 versus the control untreated cells. ^#^ *p* < 0.05 versus the same dose of chrysin. ^!^ *p* < 0.05 versus Cis 50 µg/mL.

On the mRNA level, HepG2 cells treated with cisplatin (20–100 µg/mL) (Figure 4A) or chrysin (15–60 µg/mL) (Figure 4B) for 48 h demonstrated a significant dose-dependent downregulation in GPC3 mRNA expression compared to the control untreated HepG2 cells. Moreover, co-treatment of chrysin (15–60 µg/mL) augmented the action of 50 µg/mL cisplatin on the downregulation of GPC3 mRNA expression in HepG2 cells when compared with Cis 50 µg/mL alone (*p* < 0.05, Figure 4B). 

### 2.3. Chrysin Combined with Cisplatin Decreased Sulfatase-2 (SULF2) Protein and mRNA Expressions in HepG2 Cells

**Figure 5:**
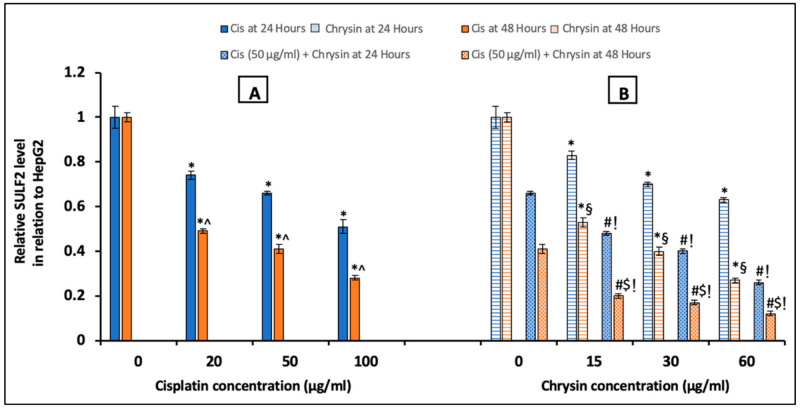
Impact of 24 and 48 h treatment with (**A**) cisplatin (Cis; 20, 50, 100 µg/mL), (**B**) chrysin (15, 30, 60 µg/mL) and combination of chrysin (15, 30, 60 µg/mL) with Cis 50 µg/mL on sulfatase-2 (SULF2) protein levels in HepG2 cells. Results are presented as Mean ± SD. * *p* < 0.05 versus the control untreated cells. ^^^ *p* < 0.05 versus the same dose of Cis treatment for 24 h. ^#^ *p* < 0.05 versus the same dose of chrysin at the same incubation time. ^!^ *p* < 0.05 versus Cis 50 µg/mL at the same incubation time. ^§^ *p* < 0.05 versus the same dose of chrysin treatment for 24 h. ^$^ *p* < 0.05 versus cis 50 µg/mL + the same dose of chrysin treatment for 24 h.

Regarding SULF2, ELISA analyses results were shown in Figure 5, in comparison to the control untreated HepG2 cells, incubation with cisplatin (20, 50, 100 µg/mL) (A) or chrysin (15, 30, 60 µg/mL) (B) alone for 24 and 48 h significantly reduced SULF2 protein levels in a dose-dependent manner. Moreover, chrysin (15–60 µg/mL) and cisplatin 50 µg/mL in combination for 24 and 48 h significantly decreased SULF2 protein levels in HepG2 cells when compared with chrysin (15-60 µg/mL) single treatment or cisplatin 50 µg/mL for 24 and 48 h, respectively (*p* < 0.05). Moreover, in the current work, a non-significant difference of SULF2 protein level was observed in case of treatment with chrysin (15–60 µg/mL) alone for 48 h when compared with both chrysin (15–60 µg/mL) and cisplatin 50 µg/mL for 24 h. However, better results were achieved when using the combination of chrysin (15–60 µg/mL) and cisplatin 50 µg/mL for 48 h compared to 24 h (*p* < 0.05, Figure 5B).

**Figure 6:**
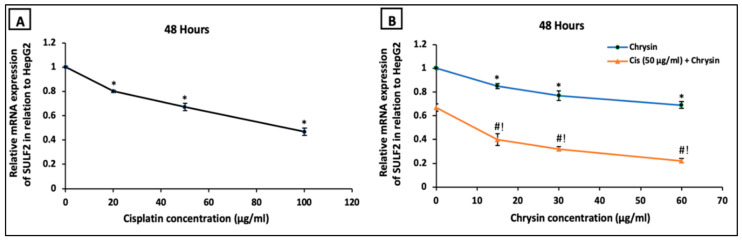
Impact of 48 h treatment with (**A**) cisplatin (Cis; 20, 50, 100 µg/mL), (**B**) chrysin (15, 30, 60 µg/mL) and combination of chrysin (15, 30, 60 µg/mL) with Cis 50 µg/mL on sulfatase-2 (SULF2) mRNA expressions of in HepG2 cells. Results are presented as Mean ± SD. * *p* < 0.05 versus the control untreated cells. ^#^ *p* < 0.05 versus the same dose of chrysin. ^!^ *p* < 0.05 versus Cis 50 µg/mL.

On the mRNA level, HepG2 cells incubated with cisplatin (20, 50, 100 µg/mL) (Figure 6A) or chrysin (15, 30, 60 µg/mL) (Figure 6B) for 48 h revealed a significant dose-dependent downregulation in SULF2 mRNA expression compared to the control untreated HepG2 cells. Moreover, co-treatment of chrysin (15–60 µg/mL) with 50 μg/mL cisplatin resulted in a more notable reduction in SULF2 mRNA expression in HepG2 cells compared to chrysin (15–60 µg/mL) single treatment or cisplatin 50 µg/mL, (*p* < 0.05, Figure 6B).

### 2.4. Chrysin Combined with Cisplatin Decreased lncRNA-AF085935 Expression in HepG2 Cells

**Figure 7:**
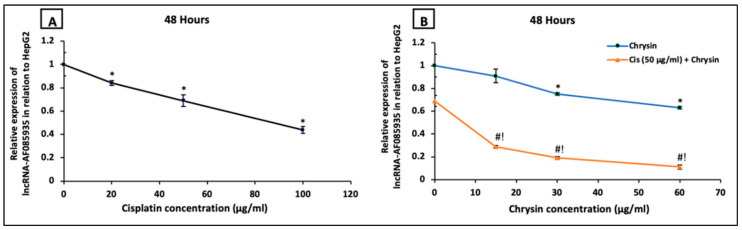
Impact of 48 h treatment with (**A**) cisplatin (Cis; 20, 50, 100 µg/mL), (**B**) chrysin (15, 30, 60 µg/mL) and combination of chrysin (15, 30, 60 µg/mL) with Cis 50 µg/mL on lncRNA-AF085935 expression in HepG2 cells. Results are presented as Mean ± SD. * *p* < 0.05 versus the control untreated cells. ^#^ *p* < 0.05 versus the same dose of chrysin. ^!^ *p* < 0.05 versus Cis 50 µg/mL.

Next, we examined if the antiproliferative action of chrysin and cisplatin on HepG2 cells involved the lncRNA-AF085935. As shown in Figure 7, HepG2 cells were treated with increasing concentrations of cisplatin (20, 50, 100 µg/mL) (A) or chrysin (15, 30, 60 µg/mL) (B) for 48 h. Except for chrysin 15 µg/mL, chrysin or cisplatin single treatment significantly reduced the lncRNA-AF085935 expression in a dose-dependent manner compared with the control untreated HepG2 cells. Furthermore, lncRNA-AF085935 expression was significantly downregulated by both chrysin (15–60 µg/mL) and cisplatin 50 μg/mL as a combination treatment when compared with chrysin (15–60 µg/mL) alone or cisplatin 50 µg/mL (*p* < 0.05, Figure 7B). 

## 3. Discussion

The HCC is a common malignancy and its management with various chemotherapeutic agents is associated with severe toxicity [34]. Thus, to minimize their toxicity, natural products have been extensively used as anticancer drugs due to their safety. However, extensive investigations were applied to determine the underlying molecular mechanism of the antiproliferative action of these natural products [20].

As tumor cells are characterized by excessive proliferation, the inhibition of proliferation is considered as a good indicator for effective antitumor therapy [35]. According to our results of the MTT assay, which was used to examine the cell viability and proliferation, the present study reported dose- and time-dependent antiproliferative activity of chrysin alone and/or in combination with cisplatin in HepG2 cells confirming that chrysin could potentiate the antitumor activity of cisplatin. In a similar way, previous reports documented the antiproliferative effect of chrysin against different human cancers, such as ovarian [36], gastric [37], and colorectal [17] cancers, in addition to hepatocellular carcinoma cells [20]. 

Moreover, it was found that GPC3, SULF2, and lncRNA-A F085935 were implicated in the proliferation of HCC with bad prognosis when overexpressed [25,33]. Therefore, our results provide a novel insight to clarify the anticancer capability of chrysin against HCC via GPC3/SULF2 axis and lncRNA-A F085935 expression. We evaluated the effect of chrysin and/or cisplatin on GPC3, a membrane-bound glycoprotein in which the protein core is covalently linked to HS GAG chains. GPC3 is known as a regulator of various growth factors and cellular pathways that modulate cellular proliferation, such as fibroblast growth factors, hedgehogs, and Wnt [38]. Wang and his colleagues, (2018), correlated GPC3 expression with liver cancer differentiation in HCC patients and reported that GPC3 promoted HepG2 cells proliferation through the hedgehog pathway [39]. Moreover, it was reported that GPC3 stimulated hepatocarcinogenesis in vitro and in vivo via stimulation of Wnt signaling [40]. 

Interestingly, GPC3 is overexpressed in HCC cells with rare expression in normal healthy cells and pathological liver cells [41]. The GPC3-immunohistochemistry has been proven as a prognostic tool for patients with HCC [26]. Capurro et al. (2005) demonstrated positive GPC3 immunolabeling in 72% of HCCs, highlighting GPC3 as a promising diagnostic marker and therapeutic target for HCC [42]. 

Indeed, many researchers proposed GPC3 as the target of effective cytotoxic agents against HCC [25,26,43]. Epigallocatechin-3-gallate, a polyphenol of the natural product green tea, in addition to sodium ascorbate, a form of vitamin C, was recently reported to exert their antiproliferative action against HCC through a marked reduction in GPC3 protein expression [25,33]. Similarly, the results of this study revealed that chrysin alone and its combination with cisplatin markedly reduced cellular GPC3 protein, immunostaining, as well as mRNA expressions in HepG2 cells, compared to untreated HepG2 cells in a dose- and time-dependent manner, confirming the involvement of GPC3 suppression as a novel antiproliferative mechanism for chrysin against HCC. 

On the other hand, it has been found that the family of heparin-degrading endosulfatases involving SULF1 and SULF2 were implicated in the pathophysiology of numerous cancers, including HCC with an opposing action either as a tumor suppressor action with SULF1 or as an oncogenic action with SULF2. It was proposed that their effects on cancer development were mediated by desulfation of HSPGs. Lai et al. (2008) explained the role of SULF2 in enhancing cell growth of HCC by desulfation of growth factor ligands that are sequestered in HSPGs storage sites in a sulfation form. Upon desulfation by SULFs, factors are released and bound to their corresponding receptors resulting in cell growth [44]. Thus, the desulfation of GPC3 by SULF2 resulted in reduction of the affinity of GPC3 for its signaling ligands with subsequent release of these ligands from GPC3 storage sites making them accessible for stimulating different pathways [25].

Moreover, the SULF2 oncogenic role was suggested to be related to the activation of several pathways, including the receptor tyrosine kinase signaling and its downstream pathways of MAPK and Akt, in addition to Wnt pathway [45]. In this context, Lai et al. (2010) reported that GPC3-dependent Wnt activation mediated the oncogenic effect of SULF2 in HCC. Desulfation of GPC3 by SULF2 released Wnt from storage sites on GPC3 followed by Wnt/β-catenin pathway activation [46]. 

Of note, SULF2 inhibitors showed chemotherapeutic benefits in HCC [25,30,47]. In agreement, treatment of HepG2 with chrysin and/or cisplatin in the present study significantly reduced SULF2 at protein and mRNA levels in dose- and time-dependent manner. Moreover, nearly similar results of GPC3 and SULF2 protein levels were observed when treating HepG2 cells with chrysin alone for 48 h or combination for 24 h confirming the chemopreventive potential of chrysin against HCC via GPC3/SULF2 axis.

Mounting evidences demonstrated involvement of lncRNAs in HCC onset, development, and metastasis. Indeed, dysregulation of various lncRNAs have been reported in HCC [48]. Among them, lncRNA–AF085935 has been strongly implicated. Motawi et al. (2019) reported elevated serum level of lncRNA–AF085935 in HCC patients, suggesting its use as a useful biomarker [49]. Interestingly, lncRNA–AF085935 is transcribed in antisense orientation with respect to GPC3. Moreover, lncRNA–AF085935 was upregulated and coexpressed with GPC3 in HCC cells and tissues [33,50]. 

Overexpression of lncRNA–AF085935 was accompanied by enhancement of HCC cell proliferation and metastasis. This effect was dependent on GPC3 upregulation. In contrast, lncRNA–AF085935 knockdown resulted in inhibition of HCC cell proliferation and migration, suggesting lncRNA–AF085935 as oncogene in HCC [51]. 

Based on the aforementioned studies, we examined if lncRNA–AF085935 is involved in the antiproliferative activity of chrysin and cisplatin on HepG2. Our results showed that chrysin and/or cisplatin markedly reduced lncRNA–AF085935 expression in HepG2 cells in a dose-dependent manner highlighting the novel role acted by chrysin in lncRNA-AF085935 regulation. This finding was consistent with a previous study that reported the antiproliferative and antiapoptotic action of epigallocatechin-3-gallate was associated with lncRNA-AF085935 suppression in HCC [33]. 

Galijatovic et al. demonstrated that chrysin is metabolized by conjugation pathways, including both glucuronidation and sulfation in both intestinal and hepatic cells [52]. Despite the beneficial bioactive effects of chrysin, its clinical application is limited owing to poor bioavailability as well as rapid metabolism and excretion. Therefore, current studies are attempting to enhance its pharmacokinetic properties via nano-modification. Of note, various in vitro studies reported anti-proliferative effect of chrysin in a time-dependent manner [53,54]. Inline, our data showed a time-dependent anti-proliferative effect of chrysin either alone or in combination with cisplatin.

In conclusion, this study is the first report to reveal that chrysin antiproliferative action against HCC could be attributed to the suppression of GPC3/SULF2 protein and mRNA expressions along with the downregulation of lncRNA-AF085935 expression. The combination of chrysin with cisplatin produced more favorable results in a dose- and time-dependent manner, suggesting that the use of chrysin as an adjuvant therapy could reduce the required dose of cisplatin and, hence, ameliorate its side effects. Further studies are required to delineate the downstream signaling ligands of GPC3 that could be targeted by chrysin in HCC.

## 4. Materials and methods

### 4.1. Drugs

Chrysin was provided as a 5 gm faint yellow powder from Sichuan Benepure Pharmaceutical Co., Ltd., Chengdu, China, while cisplatin was obtained as a 50 mL vial (1mg/mL) from Hospira, Warwickshire, UK. 

### 4.2. Cell Culture

The human hepatocellular carcinoma (HepG2) cell line was provided from the American Type Culture Collection (ATCC, Minnesota, USA) and grown using Roswell Park Memorial Institute (RPMI) 1640 culture media (Lonza Bioscience, Alpharetta, GA, USA), adding 10% fetal bovine serum, 2 mM glutamine, in addition to antibiotics of 1% penicillin/ streptomycin at 37 °C in a CO_2_ incubator. Table 1 illustrates the cell treatment protocol in which the concentrations of cisplatin and chrysin used for this study were similar to those reported before in previous studies [7,12,20,55].

### 4.3. MTT Assay for Cell Viability

The MTT cell proliferation assay (TACS^TM^ MTT, Trevigen^®^, Hagerman CT, Gaithersburg) was used for assessing cell viability according to the manufacturer’s instructions. HepG2 cells (1 × 10^3^) were placed in 96 well microplate 24 h before MTT assay. A 50 µL of serum-free media was added to cells and 10 µL of MTT solution into each well. The plate was incubated at 37 °C for 3 h. After incubation, 100 µL of detergent solution (MTT formazan) was added into each well, and then the plate was covered and incubated overnight at 37 °C. An ELISA plate reader was used to measure the color absorbance at optical density (O.D.) 590 nm, duplicate readings were taken, and then averaged for each sample. The amount of absorbance was proportional to cell number.

### 4.4. Immunocytochemistry (ICC) for the Detection of GPC3 Protein Expression

Immunocytochemistry technique was used to detect the immunopositive cells for GPC3 in the HepG2 liver cancer cell line. The cells were seeded on a 96 well plate with glass bottom followed by fixation of the cells in 100% methanol/10min at room temperature in addition to permeabilization with 0.25–0.5% Triton X-100 in PBS/10 min. After that, incubation for 24 h with GPC3 primary antibody (1:500, Abcam, Cambridge, UK) was performed. Washing after the immunostaining step was applied and secondary antibody anti-rabbit IgG (1:1000) was added for 1 h. Finally, the percentage number of GPC3 immuno-expression cells was quantified in five images/each group using the Image-Pro Plus program.

### 4.5. Enzyme-Linked Immunosorbent Assay (ELISA) for the Determination of GPC3/SULF2 Protein Levels

Cells at 1 × 10^7^ cells/mL were suspended and lysed in cell lysis buffer, and then incubation with gentle agitation was done at room temperature for up to 1 h. In cell lysate, total protein concentration was measured by the Bradford method using the Bradford protein assay kit provided by Bio Basic Inc., Ontario, Canada, according to manual instructions. The GPC3 and SULF2 protein concentration levels were assessed in cell lysate as pg/mg cell protein by using human GPC3/SULF2 ELISA kits provided from Creative Diagnostics, Shirly, NY, USA and Abbkine Scientific Co., Ltd., Wuhan, China, respectively, according to manual instructions. 

### 4.6. Quantitative Real-Time Polymerase Chain Reaction (qPCR) for the Estimation of GPC3/SULF2 mRNA Expression and lncRNA-AF085935 Expression

From cells of all groups, the total RNA was extracted with Direct-zol^TM^ RNA Miniprep Plus kits (Zymo Research Corp., CA, USA), then its quantity and quality were examined by Beckman dual spectrophotometer. The SuperScript IV One-Step RT-PCR kit (Thermo Fisher Scientific, Waltham, MA, USA) was utilized for reverse transcription of extracted RNA followed by PCR. A 48-well plate StepOne instrument (Applied Biosystems, USA) was used in a thermal profile as follows: 10 min at 45 °C for reverse transcription, 2 min at 98 °C for RT inactivation, initial denaturation by 40 cycles of 10 s at 98 °C, 10 s at 55 °C, and 30 s at 72 °C for the amplification step. After the RT-PCR run, the data were expressed in cycle threshold (Ct) for the studied genes and β-actin as a housekeeping gene. Normalization for variation in the expression of each target gene; GPC 3, SULF2, and lncRNA-AF085935 was carried out referring to the mean critical threshold (CT) expression values of β-actin by the ΔΔCt method. The relative quantitation of each gene is quantified according to the calculation of the 2^−∆∆Ct^ method. Primers sequence used for target genes was presented in Table 2. 

### 4.7. Data Analysis

The IBM SPSS software version 20 was utilized for performing the statistical analysis of this study. The results were presented as mean ± standard deviation. To evaluate the differences between groups, the one-way analysis of variance (ANOVA) accompanied by post-hoc Bonferroni correction test was applied. A p value of less than 0.05 was considered statistically significant.

## Figures and Tables

**Table 1:** Treatment protocol of HepG2 cells with different concentrations of cisplatin and chrysin at different time interval.

Group	Culture Description
Control	Untreated HepG2 cells cultured for 24 and 48 h.
Cisplatin	HepG2 cells treated with three concentrations of cisplatin (Cis; 20, 50, and 100 µg/mL) for 24 and 48 h.
Chrysin	HepG2 cells treated with three concentrations of chrysin (15, 30, and 60 µg/mL) for 24 and 48 h.
Combination	HepG2 cells treated with combined Cis 50 µg/mL and three concentrations of chrysin (15, 30, and 60 µg/mL) for 24 and 48 h.

**Table 2:** Primers sequence of the studied genes.

Genes	Forward (5′–3′)	Reverse (5′–3′)
β-actin	ATGCTCTCCCTCACGCCATC	CAGGATTCCATACCCAAGA
GPC3	GTCCCTGAACGCGACTATTT	AGCTTGTGCCAGCTCTTT
SULF2	CTGAATCCCCACATCGTCCTC	GTCCACCTTGTCATTGTCTCTCTTGT
lncRNA AF085935	CAGGGCAGCAAGGTGTTTTC	TTGGTGGGT TGCCTGATACC
